# Rapid determination of pit mud moisture content using hyperspectral imaging

**DOI:** 10.1002/fsn3.1289

**Published:** 2019-11-27

**Authors:** Min Zhu, Ping Chen, Xin‐Jun Hu, Xiang Mao, Jian‐Ping Tian, Hui‐Bo Luo, Dan Huang

**Affiliations:** ^1^ College of Bioengineering Sichuan University of Science & Engineering Zigong City China

**Keywords:** distribution map, hyperspectral imaging, moisture, pit mud, visible and near‐infrared bands

## Abstract

Lack of moisture can lead to the aging of pit mud, excessive moisture will make it difficult to maintain its shape or even collapse. Therefore, a rapid and nondestructive detection technology for moisture in pit mud using hyperspectral imaging was firstly investigated. Modeling efficiency of various processing was compared in visible (400–1,000 nm) and near‐infrared (900–1,700 nm) regions, and the optimal model was SNV‐SPA‐SVM in near‐infrared spectroscopy; the $R_{\text{pre}}^{2}$ and RMSEP of model were .9953 and 0.0029, respectively. Furthermore, the distribution map showed that the moisture in the new cellar was generally lower than that of old, and the moisture distribution of the old pit mud was more even. Moreover, the moisture content of different layers in the same cellar increased from top to bottom. This work provides strong technical support for liquor brewing enterprises to effectively implement online monitoring of pit mud changes and open a new era for the application of hyperspectral imaging technology in the field of liquor solid‐state fermentation.

## Graphical abstract

It is feasible to apply hyperspectral technology to the study of pit mud of Luzhou‐flavor liquor. Hyperspectral technology can realize rapid and nondestructive detection of pit mud moisture. The optimal model for pit mud moisture is SNV‐SPA‐SVM in near‐infrared spectroscopy.

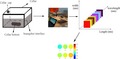

## 1 INTRODUCTION

As one of the six distilled liquor in the world, Chinese baijiu is an invention that full of creative wisdom and cultural charm in China. The production and market share of Luzhou‐flavor liquor account for about 70% of the entire liquor industry (Hu, 2015). As the foundation for the brewing of Luzhou‐flavor liquor, pit mud determines the quality of product to a large extent. The water in pit mud dissolves various organic matter and mineral nutrients and acts as a transporting substance. A series of biochemical reactions in the microbial organism is also inseparable from water (Zhang et al., 2013). Therefore, the lack of water will lead to mud hardening, the precipitation of salt, and the death of some microbes in pit mud, which will eventually cause the degradation of pit mud, thus seriously affect the production (Jing, Tang, Ren, & Yao, 2010). However, the excessive moisture will result in that it is hard to maintain shape even the collapse of the pit. Therefore, the moisture of the mud is regarded as a crucial detection indicator in the evaluation standard of the Luzhou‐flavor liquor.

At present, there have been many reports on the moisture determination in pit mud. For example, Xie et al. (2018) compared the difference between moisture content in different regions and quality levels and studied its distribution features. Zhang et al. (2014) found that moisture, pH, and humus had a great influence on the quality of the mud based on the analytic hierarchy process. Liu et al. (2018) found that moisture, total nitrogen, and ammonium nitrogen increased with the improvement of the quality of the mud via correlation analysis, the pH value was close to 7. However, all the above method for the determination of the mud moisture is natural drying method, which is destructive, time‐consuming, labor‐intensive, etc., and the results often lag behind the actual production, which cannot meet the relevant staff to the rapid and nondestructive online assessment of mud.

Therefore, hyperspectral imaging emerged as a rapid, nondestructive, advanced optical technology. Hyperspectral imaging is an image data technology based on a very large number of narrowband. It combines imaging with spectroscopy technology and simultaneously detects the spectral and spatial information, which is closer to the real properties of the object. It has attracted countless researchers as a powerful new tool for lossless online testing a variety of products (Li, Rao, & Ying, 2011). At present, hyperspectral technology has been widely implemented in nondestructive testing of agriculture products, such as apple (Baranowski, Mazurek, & Pastuszka‐Woźniak, 2013), cucumber (Cen, Lu, Zhu, & Mendoza, 2016), orange (Li et al., 2011), peach (Pan et al., 2016), potatoes (Cho et al., 2013), cocoa beans (Caporaso, Whitworth, Fowler, & Fisk, 2018), fish (Sivertsen, Heia, Hindberg, & Godtliebsen, 2012), beef (ElMasry, Sun, & Allen, 2012, 2013), pork (Barbin, ElMasry, Sun, & Allen, 2012, 2013), and cereal crops (Caporaso, Whitworth, & Fisk, 2018; Orina, Manley, & Williams, 2017; Vigneau, Ecarnot, Rabatel, & Roumet, 2011). Sun et al. (2017) established a quantitative prediction model of chlorophyll content in peaches using hyperspectral imaging and quickly distinguished the degree of decay of peaches. Zou et al. (2011) rapidly detected the chlorophyll content of cucumber leaves, and a visual distribution map was drawn using hyperspectral technology. ElMasry, Sun, and Allen (2013) used hyperspectral imaging to quickly detect fat and protein content in beef and visualize their distribution to compare the differences between various beef samples. Moreover, this technology can also be used to evaluate indicator changes in solid‐state fermentation. Zhu et al. (2016) used hyperspectral to investigate the moisture and total acid content of vinegar, and the results showed that it was feasible to determine changes in the main indicators during solid‐state fermentation using hyperspectral imaging. However, there has been no report on the application of hyperspectral technology to the solid‐state brewing of liquor, and research in this field has been blank.

**Figure 1:**
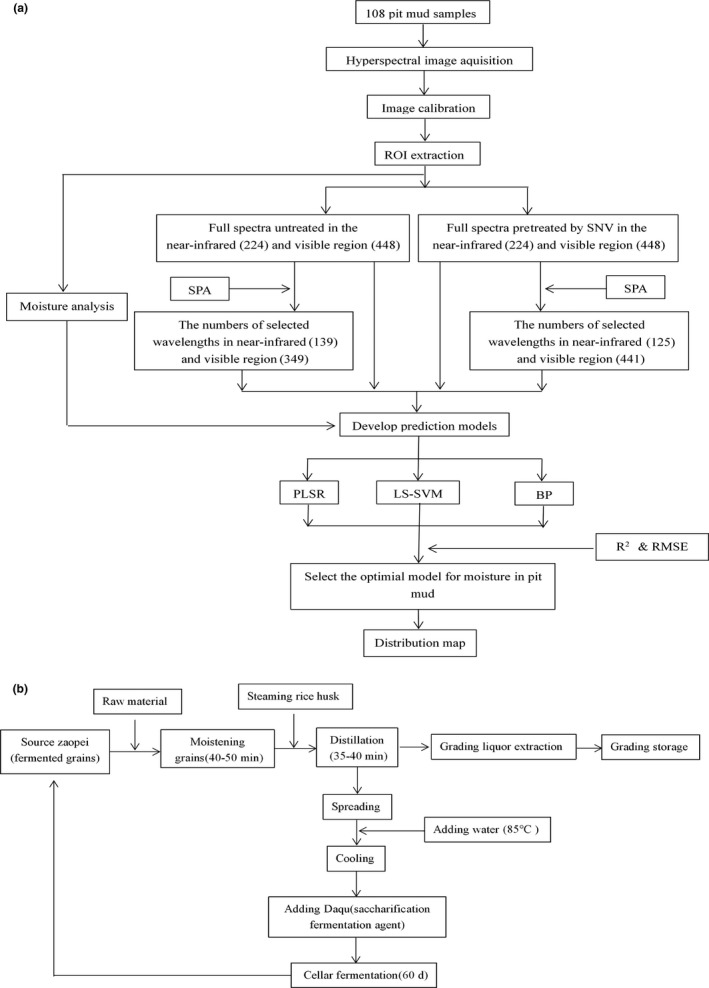
Flowchart of the experiment procedure and Luzhou‐flavor liquor production process. (a) Flowchart of the experiment procedure to develop an optimal model for detection of pit mud moisture using hyperspectral imaging. (b) Flowchart of Luzhou‐flavor liquor production process

Due to preliminary experiments and literature review showed that O‐H of moisture has characteristic absorption from visible to near‐infrared region even interfere with signals from other compounds. Consequently, the objective of this work is to establish rapid prediction models of pit mud moisture based on near‐infrared (900–1,700 nm) and visible spectroscopy (400–1,000 nm), untreated and standard normal variate (SNV) pretreated, full and characteristic spectrum, and three modeling methods including partial least squares regression (PLSR), least squares support vector machine (LS‐SVM), and back‐propagation network (BP). A total of twenty‐four models were acquired. The optimal model was selected through the determination coefficients ($R_{\text{cal}}^{2}$ and $R_{\text{pre}}^{2}$) and root mean square error (RMSEC and RMSEP) of calibration and prediction set, and the moisture distribution difference of various pit mud samples was visualized based on the obtained optimal model (Figure 1a). This work provides strong technical support for the transformation and upgrading of liquor brewing industrialization and intelligent real‐time online monitoring.

## 2 MATERIALS AND METHODS

### 2.1 Pit mud samples

**Figure 2:**
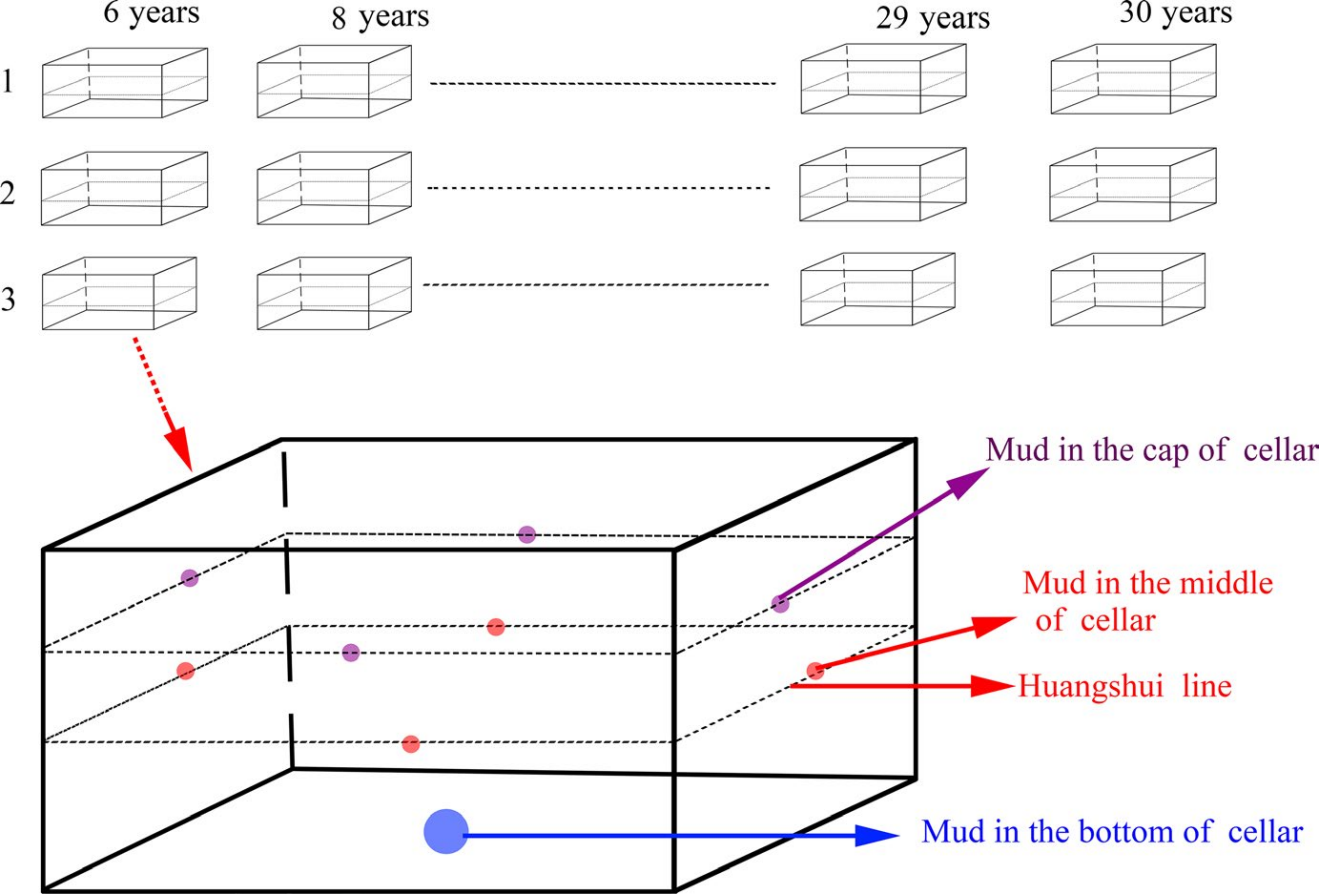
A diagram of the sampling method

The original pit mud samples were collected in June 2019 and provided by Yibin Jinxilai Liquor Co., Ltd, located in the south‐central of Sichuan Province (26°03′–34°19′ northern latitude, 97°21′–108°31′ east longitude). The production process flowchart of Luzhou‐flavor liquor is shown in Figure 1b. After fermentation, the fermented grains were removed in the cellar; the pit mud around the same layer was collected and fully mixed to form a sample. The sampling sites of the same pit include three layers of upper, middle, and lower, that is, cellar cap, huangshui line, and cellar bottom, so three samples were obtained from the same pit. Finally, a total of 108 pit mud samples were obtained from 36 pits with 12 years (6, 8, 9, 12, 15, 18, 21, 23, 24, 27, 29, and 30), in which each pit age corresponds to No. 1, 2, and 3 pits. The specific sampling method is shown in Figure 2. Collected samples were placed in ice box and transported back to the laboratory for rapid experiments.

The 108 samples were assigned to the calibration set and prediction set with the method of Kennard–Stone (K‐S) algorithm according to a proportion of 2:1. Therefore, 72 samples were selected as calibration set for developing the calibration model, whereas the remaining 36 samples were used as prediction set to verify the prediction performance of the calibration model.

### 2.2 Hyperspectral imaging system and data acquisition

**Figure 3:**
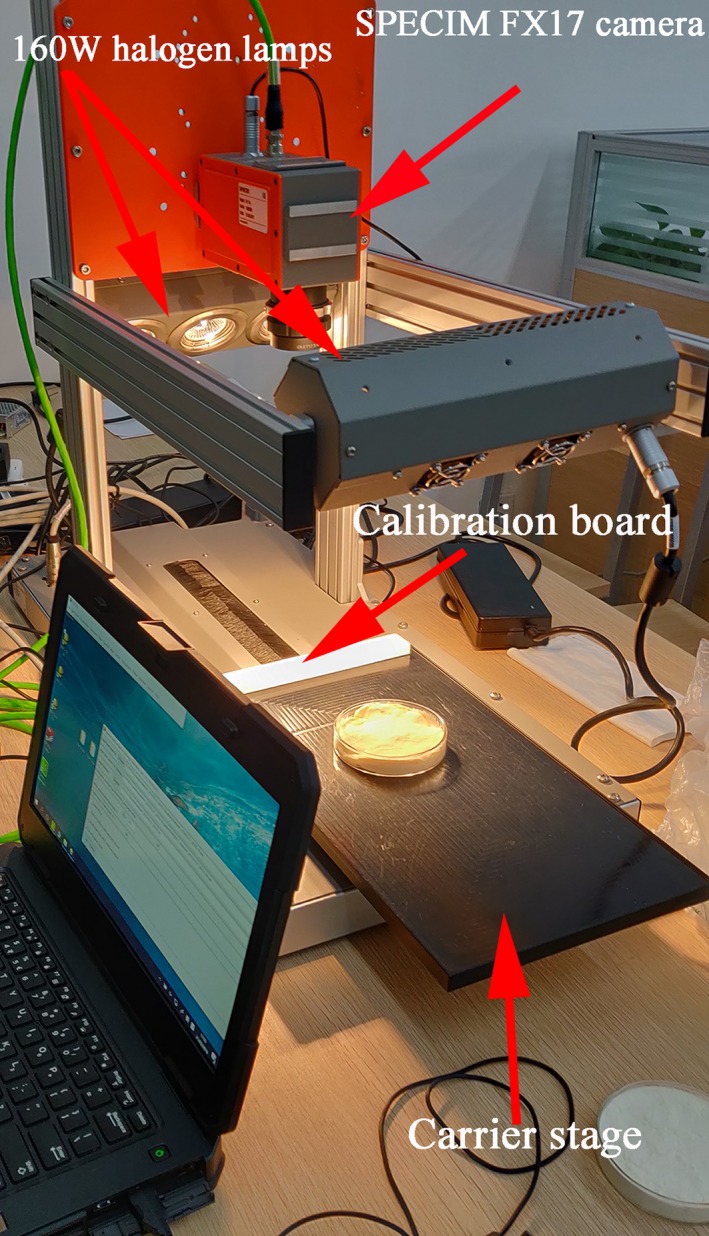
Structural diagram of the hyperspectral imaging system

Hyperspectral images of samples were obtained using a hyperspectral imaging system in reflection mode. The system (Figure 3) consisted of a SPECIM FX17 series hyperspectral camera connected to an imaging spectrometer in Finland, two sets of 160W Y‐type optical fiber halogen lamps (3900ER, Illumination Technologies Inc), a precision electronically controlled carrier stage (IRCP0076 of‐ICOMB001, Isuzu), and LUMO Scanner software (Spectral Image, Isuzu). The parameters of near‐infrared and visible region were set, respectively, as shown in Tables 1 and 2, including peak lighting, exposure frequency, time, and scanning speed. Considering the strong reflectivity of glassware, the quartz‐based glassware was more suitable for spectral data acquisition during the experiment to improve the accuracy of the model. Under the set parameter environment, quartz vessel filled with pit mud and paved was placed in the center of the carrier platform. The hyperspectral original image information of 108 pit mud samples was collected at 400–1,000 and 900–1,700 nm region, respectively. Thus, raw spectral data of 224 wavelengths in the near‐infrared region and 448 wavelengths in the visible region were obtained, respectively.

**Table 1:** Parameter setting in near‐infrared region

Calibration board type	Beige board
Beam peak	2,112
Shutter	Opened
Frame time (Hz)	50 Hz
Exposure time (ms)	4.02
Spectral binning	1
Spatial binning	1
Trigger mode	Internal
Scanning speed (mm/s)	16.42
Starting and ending position of object	Object start: 94.48 Object stop: 141.43
Starting and ending position of board	Board start: 22.07 Board stop: 32.04
Dark subtraction	Shutter
White reference	Manual
Red band	Band 31 (1,039.53 nm)
Green band	Band 76 (1,196.37 nm)
Blue band	Band 131 (1,389.61 nm)

**Table 2:** Parameter setting in visible region

Calibration board type	Gray board
Beam peak	1,024
Shutter	Opened
Frame time (Hz)	50 Hz
Expctral time (ms)	8.00
Spectral binning	1
Spatial binning	1
Trigger mode	Internal
Scanning speed (mm/s)	10.79
Starting and ending position of object	Object start: 94.48 Target stop: 141.43
Starting and ending position of board	Board start: 22.07 Board stop: 32.04
Dark subtraction	Shutter
White reference	Manual
Red band	Band 191: 650.96 nm
Green band	Band 117: 551.57 nm
Blue band	Band 55: 469.28 nm

### 2.3 Hyperspectral imaging processing

The hyperspectral data collected by the camera are a three‐dimensional data cube; each pixel contains a full‐band spectral curve. Apart from sample information, there are also interference signals, such as dark current, high‐frequency random noise in the process of spectral data acquisition. The region of interest (ROI) with 100 × 100 pixels was manually selected from the raw spectra, which should be fairly flat and representative. The average spectral values of each pixel in the ROI were calculated to obtain the data before correction. Because of the nonuniformity of fermentation status, the growth and metabolism of microbes in pit mud vary greatly, and the spectral values of each pixel will be different under different illumination intensity. To eliminate the systematic error caused by nonuniform illumination, the spectral values were converted into spectral reflectivity via black‐and‐white correction processing, removing the influence of illumination intensity changes.

Calibration board should be used for black‐and‐white correction, considering that the color difference between the samples and board was too large; it was easy to interfere with the collection of spectral data. Therefore, calibration boards which were similar to a specific sample and slightly better in spectral reflectance performance were chosen. Moreover, due to the difference between near‐infrared and visible region, a gray Teflon board in the visible region and a beige Teflon board in the near‐infrared region were chosen. The dark reflection image was obtained via covering the camera lens with an opaque cap, and a white reflection image was obtained using a Teflon gray and beige board. The corrected relative reflectivity (*R*) was calculated according to the following equation:

(1) $$R=\frac{I-D}{W-D}$$

where *I* is the original hyperspectral image, *D* is the dark image, and *W* is the white reflectance image. The corrected spectral reflectivity was used for further analysis.

### 2.4 Determination of moisture content in pit mud

The specific steps of moisture determination in pit mud were as follows:

Took clean weighing bottle and placed it in the drying box at 101–105°C. The cap was obliquely supported on the edge of the bottle, heated for 1.0 hr, removed and cooled for 0.5 hr in the dryer. Weighed and dried it repeatedly until the quality difference between the two times was no more than 2 mg, that is, constant weight.

Weighing 2–10 g sample (accurate to 0.0001 g), put into weighing bottle, the height of the sample was not more than 5 mm, capped after precise weighing, placed in a 101–105°C drying box, the cap was slanted to the edge of the bottle, after 2–4 hr of drying, capped and removed bottle, weighed after cooled in the dryer for 0.5 hr. Then, put it in 101–105°C drying box for about 1 hr, continue to take it out, cooled in the dryer for 0.5 hr, and weighed them again. Repeated the above operation until the quality difference between the two times was no more than 2 mg, that is, constant weight. Moisture content (*X*) in samples was calculated according to the following equation:

(2) $$X=\frac{\left(m_{1}-m_{2}\right)}{\left(m_{1}-m_{3}\right)}\times 100$$

where *m*_1_ is the quality of weighing bottles and samples, *m*_2_ is the quality of weighing bottles and samples after drying, *m*_3_ is the quality of weighing bottle, 100 is the conversion factor. Moisture in pit mud was used for further analysis.

### 2.5 Data processing and analysis

**Figure 4:**
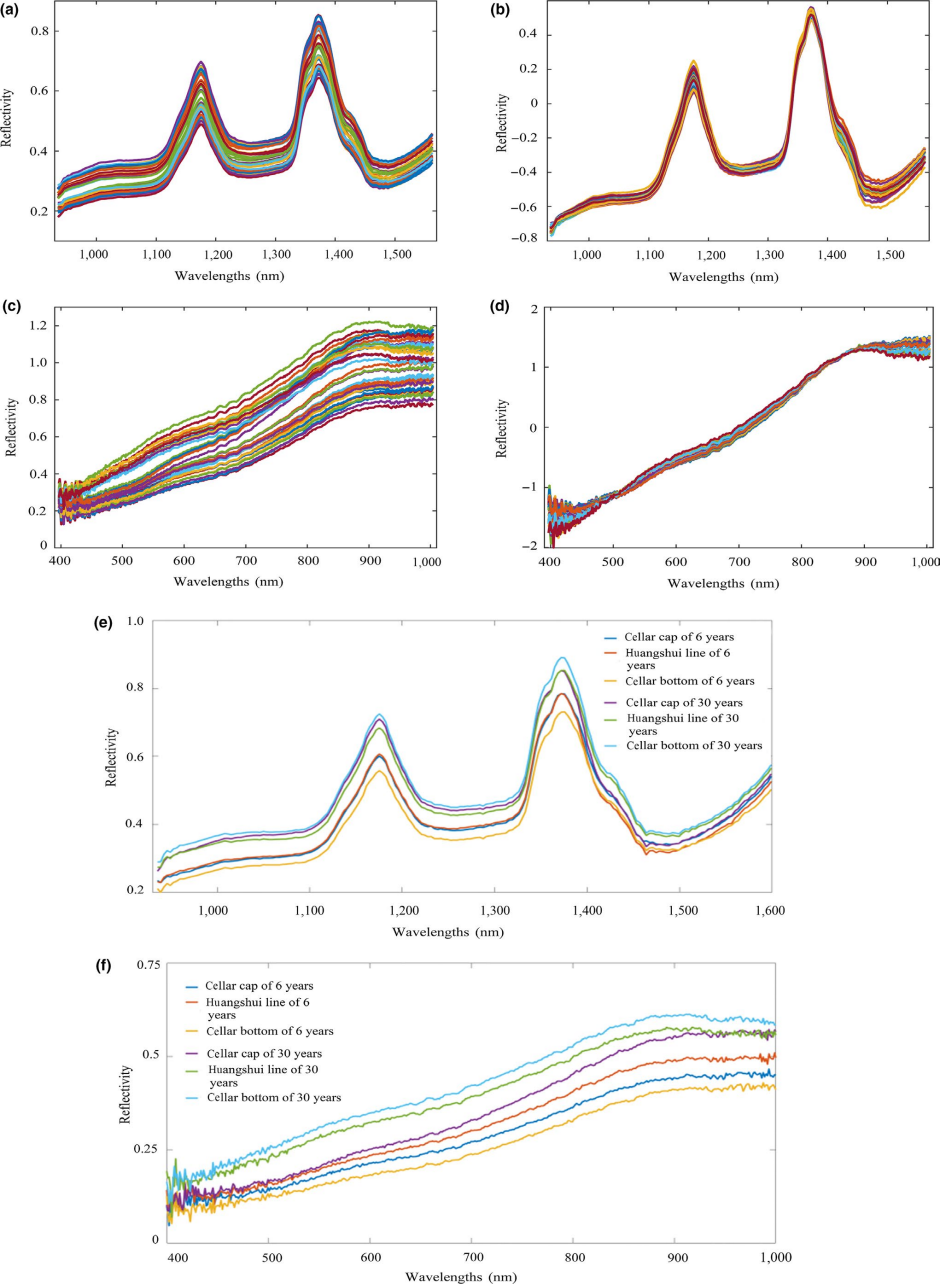
Spectral reflection curves of pit mud samples. (a) Original spectral reflection curves in the near‐infrared region. (b) Pretreated spectral reflection curves in the near‐infrared region. (c) Original spectral reflection curves in the visible region. (d) Pretreated spectral reflection curves in the visible region. (e) Spectral reflection curves of pit mud samples of 6 and 30 years in near‐infrared spectroscopy. (f) Spectral reflection curves of pit mud samples of 6 and 30 years in visible spectroscopy

Considering that noise, baseline variation and others may affect the accuracy of the model in the data acquisition process; SNV can effectively remove high‐frequency noise (Munera et al., 2017; Yan et al., 2017), prevent baseline drift, and optimize spectral signals. Thus, original spectral information was preprocessed by SNV, which means that spectra were corrected mainly using mean and variance of samples to eliminate the influence of spectral linear translation, which was shown in Figure 4a,b,c,d. Spectral reflectivity (X*_i_*_(SNV)_) in samples was calculated according to the following equation:

(3) $$X_{i(\text{SNV})}=\frac{X_{i}-\bar{X_{i}}}{\sigma_{i}}$$

(4) $$\bar{X_{i}}=\frac{1}{m}\sum_{j=1}^{m}X_{i,j}$$

(5) $$\sigma_{i}=\sqrt{\frac{1}{m}\sum_{j=1}^{m}{\left(X_{i,j}-\bar{X_{i}}\right)}^{2}}$$

where $\bar{X_{i}}$ is the spectral mean value of samples, *σ*_i_ is the spectral standard deviation of samples.

O‐H was selective for spectral absorption, whereas the spectral signals produced by the sample were the overlap of various substances in pit mud. To improve the robustness and prediction capability of the model, it was indispensable to find feature‐related wave bands and eliminate irrelevant. The successive projections algorithm (SPA) was used to select characteristic wave bands. SPA generalized the spectral information of most samples by selecting a few wavelengths in the raw spectral to maximize the avoidance of information redundancy.

Partial least squares regression, LS‐SVM, and BP three models were established based on the spectral data of near‐infrared, visible, untreated and pretreated, full spectrum, and characteristic spectrum, respectively, combined with the measured values of pit mud moisture, a total of 24 models. The optimal model was obtained by comparing the *R* ^2^ and RMSE of the calibration set and prediction set of the models.

Region of interest (100 × 100) was selected in the image, and each pixel in the region was regarded as a sample. The characteristic spectral data corresponding to each sample were brought into the optimal prediction model to calculate the moisture prediction value of all the pixels in the region and then mapped the predicted value to 0–255 for graying; the gray value of each pixel was obtained. Finally, the pseudocolor processing was carried out to acquire the visual images of moisture content in pit mud.

## 3 RESULTS AND DISCUSSION

### 3.1 Moisture content analysis

The statistics of the moisture content measured by the conventional analysis method is listed in Table 3. It can be seen that wide variations in moisture contents ranging from 30% to 40% were obtained, which was the basic data for the establishment of calibration model.

**Table 3:** Statistics of moisture measured by the conventional analysis method

Different years	Calibration set			Prediction set		
	No. of samples	Range (%)	Mean ± *SD* (%)	No. of samples	Range (%)	Mean ± *SD* (%)
6	6	33.8–36.9	35.3 ± 2.2	3	34.3–36.0	35.2 ± 2.1
8	6	32.6–36.4	34.9 ± 3.0	3	34.1–36.8	35.7 ± 2.7
9	6	35.9–37.9	36.7 ± 2.1	3	36.5–38.1	37.0 ± 1.7
12	6	32.5–36.7	34.5 ± 1.9	3	34.2–36.9	35.5 ± 1.3
15	6	34.1–37.0	36.5 ± 1.7	3	35.9–37.8	36.2 ± 1.5
18	6	32.9–37.5	35.2 ± 2.2	3	34.5–37.0	36.1 ± 2.0
21	6	34.3–39.7	37.1 ± 3.9	3	35.7–38.3	37.7 ± 2.3
23	6	35.1–38.5	36.7 ± 3.1	3	34.3–35.7	35.0 ± 2.8
24	6	34.9–38.8	37.6 ± 2.9	3	34.3–39.9	37.3 ± 2.0
27	6	34.1–37.3	35.8 ± 2.8	3	34.7–36.1	35.5 ± 3.7
29	6	36.8–39.7	37.9 ± 1.8	3	37.0–38.6	37.5 ± 2.3
30	6	38.3–40.6	40.1 ± 3.3	3	38.7–40.1	39.4 ± 3.0

### 3.2 Spectral analysis of moisture in pit mud

In the whole process of liquor brewing, pit‐entry fermentation is a very important process, in which pit mud plays a key role. After decades or even hundreds of years of fermentation, the pit mud contains abundant microbes and metabolites. Hyperspectral imaging technology can quickly detect the main variables of pit mud, explore the fermentation status of microbes via spectral information, and find out the essential differences of the cellar at various layers and ages.

For the convenience of observation, taking pit mud of two ages as an example, the spectral reflectance curves of pit mud of different layers and ages in near‐infrared and visible spectroscopy were described. There were six curves including near‐infrared full spectrum (224 wavelengths) and visible full spectrum (448 wavelengths), respectively, as shown in Figure 4e,f; one was a new cellar for 6 years, and the other was an old cellar for 30 years. The spectral reflectance of pit mud in 30 years was generally higher than that in 6 years, and the spectral reflectance curves of pit mud at different layers were also different, which indicates that hyperspectral imaging can identify pit mud at different layers and years.

### 3.3 Feature‐related wavelengths selection

**Figure 5:**
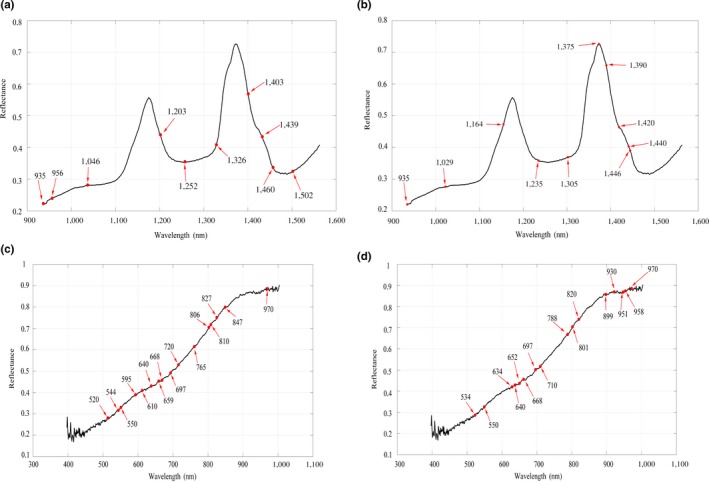
Wave bands correlated with moisture in the near‐infrared and visible region. (a) Distribution of wave bands correlated with moisture in pretreated average spectrum in near‐infrared spectroscopy. (b) Distribution of wave bands correlated with moisture in untreated average spectrum in near‐infrared spectroscopy. (c) Distribution of wave bands correlated with moisture in pretreated average spectrum in visible spectroscopy. (d) Distribution of wave bands correlated with moisture in untreated average spectrum in visible spectroscopy

To compare and analyze the performance of full spectrum and characteristic spectrum modeling, it was essential to select wavelengths closely related to moisture in pit mud and to eliminate the influence of irrelevant wavelengths. To determine the optimal number of variables, the effects of compounds other than moisture and the amount of calculation of the full spectrum (224 bands from 900 to 1,700 nm and 448 bands from 400 to 900 nm) should be taken into account. Because of the difference of spectral data obtained from visible and near‐infrared range, untreated and SNV pretreated, four groups of characteristic wavelengths were acquired via SPA: 349 and 441 bands were obtained in the visible region, whereas 139 and 125 bands were obtained in the near‐infrared region, including untreated and pretreated. Thus, we can know that the numbers of characteristic wavelengths after SPA screening were still large. To clearly show the absorption range correlated with pit mud moisture, we selected four groups of wavelengths with higher frequency to display, respectively, as shown in the Figure 5. The result shows that the characteristic peak of moisture was mostly concentrated below 1,500 nm, both strong absorption in visible and near‐infrared spectroscopy, which had similar to that of some study (Workman, 2007).

### 3.4 Prediction of moisture in pit mud

Pre‐experiment with different gradients was carried out to find the characteristic absorption peak of moisture in pit mud; the result showed that feature‐related wave bands were within 1,500 nm, which was verified by literature (Workman, 2007). Therefore, in this paper, the spectral information corresponding to the front 180 wavelengths was selected for the modeling of near‐infrared spectroscopy. Different quantitative prediction models (PLSR, SVM, BP) were established for calibration set samples based on various mathematical algorithms, totaling 24 models. The optimal prediction model was evaluated and screened through the *R* ^2^ and RMSE of the calibration set and prediction set. The SVM model was generally superior to the other two models in calibration and prediction set, regardless of the robustness or predictive performance of the model; thus, it was considered as a relatively good model (Table 4).

**Table 4:** Statistical table of performance of fitting models

Region	Pretreatment or not	Spectral range	Prediction models	Calibration set		Prediction set	
				$R_{\text{cal}}^{2}$	RSMEC	$R_{\text{pre}}^{2}$	RSMEP
In near‐infrared	Untreated	Full spectrum	PLSR	.9953	0.0026	.9717	0.0067
			SVM	.9581	0.0078	.9260	0.0108
			BP	.6278	0.0232	.6180	0.0246
		SPA	PLSR	.9933	0.0031	.9833	0.0051
			SVM	.9524	0.0083	.9303	0.0105
			BP	.9396	0.0152	.9402	0.0152
	SNV	Full spectrum	PLSR	.9958	0.0024	.9678	0.0074
			SVM	.9994	0.0009	.9936	0.0033
			BP	.3998	0.0291	.4549	0.0303
		SPA	PLSR	.9927	0.0032	.9721	0.0069
			SVM	.9995	0.0008	.9953	0.0029
			BP	.7805	0.0193	.8002	0.0191
In visible	Untreated	Full spectrum	PLSR	.9891	0.0040	.9281	0.0109
			SVM	.9998	0.0005	.9709	0.0069
			BP	.6829	0.0217	.4644	0.0291
		SPA	PLSR	.9909	0.0037	.9396	0.0100
			SVM	.9991	0.0034	.9991	0.0040
			BP	.9971	0.0064	.9968	0.0072
	SNV	Full spectrum	PLSR	.9989	0.0013	.9830	0.0052
			SVM	.9978	0.0018	.9948	0.0029
			BP	.6008	0.0247	.5742	0.0258
		SPA	PLSR	.9985	0.0015	.9902	0.0039
			SVM	.9992	0.0011	.9709	0.0018
			BP	.6821	0.0219	.7279	0.0207

Next, on the basis of SVM model, the modeling effects of untreated and SNV pretreated spectral data were compared. From Table 3, we can see that in near‐infrared region, the modeling effect of SNV pretreatment was always better than that of untreated spectral data in both full and characteristic bands. Moreover, in the visible range, the pretreated data were significantly better than the untreated on the basis of the full spectrum. In the characteristic spectrum, the untreated model had better generalization performance, but the error was larger. Therefore, the SNV pretreatment was chosen to get rid of noises and prevent the baseline drift.

Further compared and analyzed the performance of the SVM model in near‐infrared and visible regions, from Table 3, it can be concluded that the modeling effect of near‐infrared spectral data was always better than that of visible range, whether in full or characteristic spectrum.

**Figure 6:**
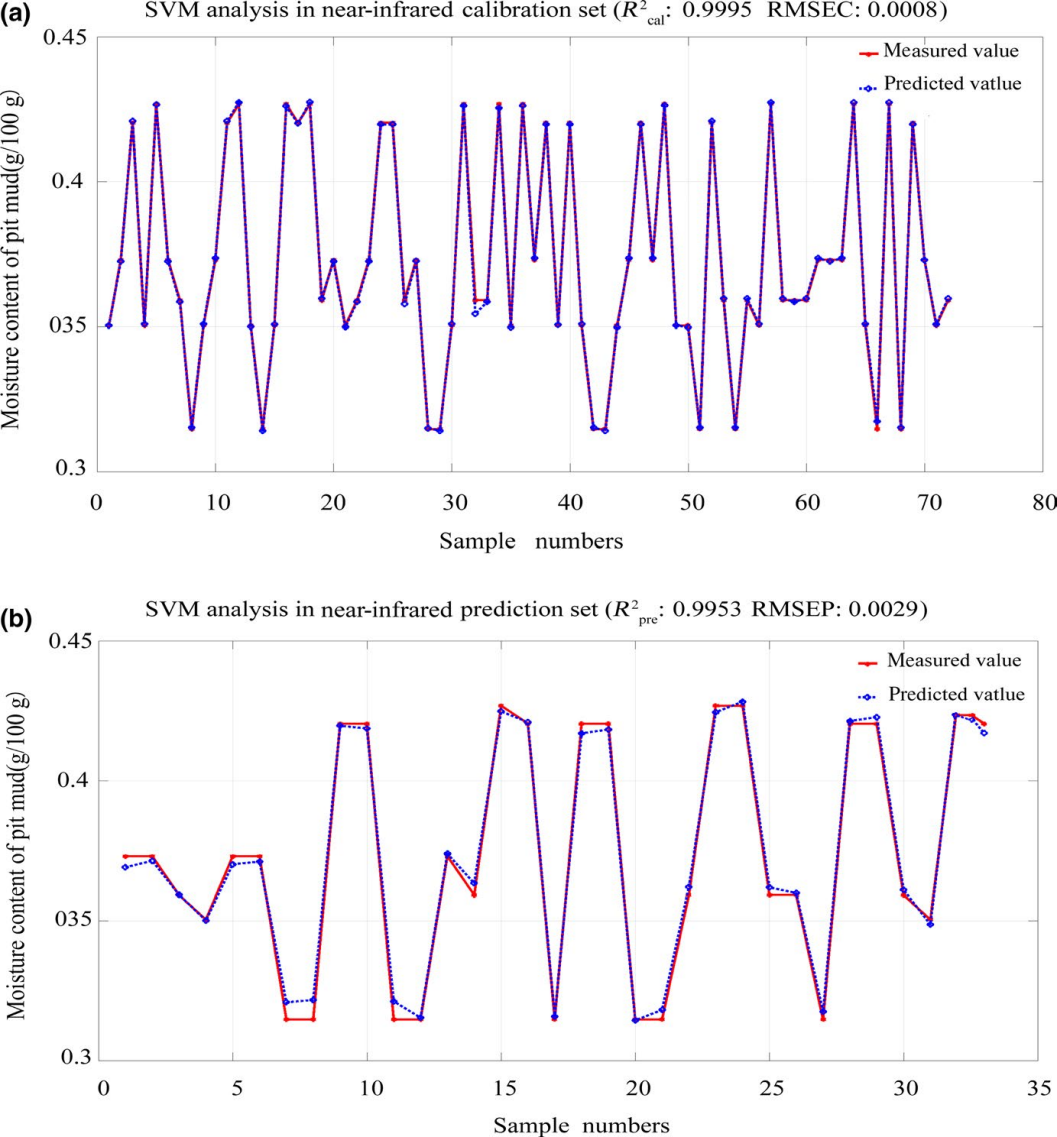
Effect figure of SNV‐SPA‐SVM model based on near‐infrared spectroscopy. (a) Effect figure of pit mud moisture model on calibration set. (b) Effect figure of pit mud moisture model on prediction set

Finally, compared the model performance of full spectrum and characteristic spectrum, the SVM model based on characteristic spectrum had higher accuracy, smaller error, and better prediction effect than that of characteristic (Table 3). Furthermore, taking a large amount of data in full spectrum modeling and the difficulty in processing into account, large‐scale servers were needed. Consequently, under the condition of guaranteeing the accuracy of the model, modeling with characteristic variables was preferred to reduce the computational load and improve the work efficiency. Therefore, the optimal model for quantitative prediction of pit mud moisture was SNV‐SPA‐SVM model based on near‐infrared spectroscopy (Figure 6), The $R_{\text{cal}}^{2}$ of calibration set was .9995, the RMSEC was 0.0008, the $R_{\text{pre}}^{2}$ of prediction set was .9953, and the RMSEP was 0.0029.

### 3.5 Visualization of moisture content

**Figure 7:**
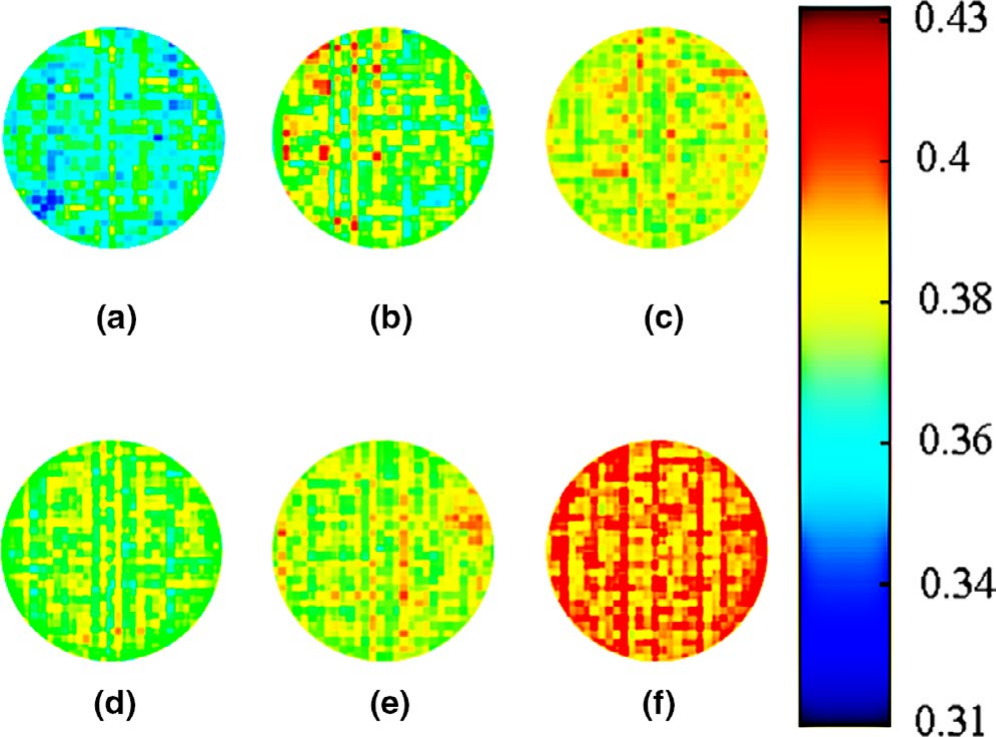
The distribution map of moisture in pit mud of 6 and 30 years. (a) Pit mud of upper layer for 6 years. (b) Pit mud of middle layer for 6 years. (c) Pit mud of bottom layer for 6 years. (d) Pit mud of upper layer for 30 years. (e) Pit mud of middle layer for 30 years. (f) Pit mud of bottom layer for 30 years

The spectral data of each pixel in the ROI were brought into the optimal model to calculate the predicted value of pit mud moisture content, and then, pseudocolor processing was carried out to obtain the visual distribution map (Figure 7). The result shows that the moisture in the cellar of 6 years was generally lower than that of 30 years, the new cellar was about 36%–38% and the old was about 37%–40%, and the moisture distribution of the old pit mud was more even compared with the new pit. This may be due to the long‐term succession of microbial communities, which resulted in the accumulation of a large number of nutrients in pit and increased water holding capacity of pit mud. Moreover, the moisture content of the same cellar increased from top to bottom in turn.

## 4 CONCLUSION

Pit mud, as the foundation of Luzhou‐flavor liquor brewing, plays a decisive role in quality and liquor yield. Moisture content was a crucial factor for the normal metabolism of beneficial microbes in pit mud. In this work, a rapid and nondestructive detection method for moisture in pit mud using hyperspectral imaging was investigated. Raw spectral data of 108 pit mud samples in near‐infrared and visible range were collected. SNV was used to remove noise and prevent baseline variation, which may reduce the accuracy of the model. SPA was used to select characteristic wavelengths correlated with moisture. Twenty‐four quantitative prediction models were developed for calibration samples based on full spectrum and specified spectrum; according to the *R* ^2^ and RMSE of the calibration and prediction set, SNV‐SPA‐SVM in near‐infrared region was considered as the optimal prediction model. Based on this model, the visual distribution map of pit mud moisture was obtained. The result shows that the moisture content in the new cellar was generally lower than that of old, and the moisture of the same cellar increased from top to bottom in turn. The cloud image can visually display the moisture distribution of pit mud and help to judge the complex fermentation state, thus providing a reference for the online monitoring of pit fermentation.

Consequently, a rapid prediction method for pit mud moisture content was firstly established, which opens the door for the application of hyperspectral imaging technology in liquor solid‐state field, and also injects new vigor into the transformation and upgrading of industrialization of Chinese baijiu.

## CONFLICT OF INTEREST

The authors declare that they do not have any conflict of interest.

## ETHICAL STATEMENT

This study does not involve any human or animal testing.
